# Secondary Metabolites Found among the Species *Trattinnickia rhoifolia* Willd

**DOI:** 10.3390/molecules26247661

**Published:** 2021-12-17

**Authors:** Agerdânio Andrade de Souza, Brenda Lorena Sánchez Ortíz, Rosemary de Carvalho Rocha Koga, Priscila Faimann Sales, Divino Bruno da Cunha, Ana Luiza Mantovaneli Guerra, Gisele Custódio de Souza, José Carlos Tavares Carvalho

**Affiliations:** 1 Post-Graduate Program in Pharmaceutical Innovation, Pharmacy Course, Department of Biological and Health Sciences, Federal University of Amapá, Rodovia Juscelino Kubitschek, km 02, Macapá CEP 68903-419, Amapá, Brazil; agerdanio.souza@unifap.br (A.A.d.S.); rosemarykoga@gmail.com (R.d.C.R.K.); pfaimann@gmail.com (P.F.S.); custodio_gisele@yahoo.com.br (G.C.d.S.); 2 Indigenous Intercultural Licensing Course, Binational Campus, Federal University of Amapá, Rodovia BR 156, n° 3051, Universidade, Oiapoque CEP 68980-000, Amapá, Brazil; 3 Research Laboratory of Drugs, Department of Biological and Health Sciences, Federal University of Amapá, Rodovia Juscelino Kubitschek, km 02, Macapá CEP 68903-419, Amapá, Brazil; charmed1797@gmail.com; 4 Department of Biological and Health Sciences s/n, Institute of Xingu Studies, Federal University of Southern and Southeastern Pará, Centro, São Félix do Xingu CEP 68380-000, Pará, Brazil; divinobruno@unifesspa.edu.br; 5 Veterinary Medicine Course, Center of Rural Sciences, Federal University of Santa Maria, Avenida Roraima, 1000, Camobi, Santa Maria CEP 97105-900, Rio Grande do Sul, Brazil; serioususerana@gmail.com

**Keywords:** *Burseraceae*, pharmacology, phytochemistry, review, *Trattinnickia*

## Abstract

Plant-derived products may represent promising strategies in the treatment of Neglected Tropical Diseases (NTDs). From this perspective, it is observed that the Amazon phytogeographic region contains the tribe *Canarieae* of the *Burseraceae* family, composed of trees and shrubs supplied with resin channels. Its uses in folk medicine are related to aromatic properties, which have numerous medicinal applications and are present in reports from traditional peoples, sometimes as the only therapeutic resource. Despite its economic and pharmacological importance in the region, and although the family is distributed in all tropical and subtropical regions of the world, most of the scientific information available is limited to Asian and African species. Therefore, the present work aimed to review the secondary metabolites with possible pharmacological potential of the species *Trattinnickia rhoifolia* Willd, popularly known as “*Breu sucuruba*”. To this end, an identification key was created for chemical compounds with greater occurrence in the literature of the genus *Trattinnickia*. The most evident therapeutic activities in the consulted studies were antimicrobial, antioxidant, anti-inflammatory, antiviral, antifungal, anesthetic and antiparasitic. An expressive chemical and pharmacological relevance of the species was identified, although its potential is insufficiently explored, mainly in the face of the NTDs present in the Brazilian Amazon.

## 1. Introduction

Over the years, natural products have been used as raw material sources in the synthesis of chemical compounds that result in new drugs, a good part of which are used in the treatment of Neglected Tropical Diseases (NTDs) [1]. That is where the drugs in clinical use come from, corresponding to 40% of the drugs currently available, which are directly or indirectly derived from natural sources in the rainforest [2]. In this sense, Brazilian biodiversity is considered an extremely rich source of bioactive substances, highly diversified in innovative chemical structures [3], that dictate the planning of new chemical syntheses.

In parallel to the biological variety found in the Amazon Forest, there are NTDs that encompass a group of 17 common infectious diseases in the region, including dengue, rabies, trachoma, buruli ulcer, endemic treponematosis, Hansen’s disease (leprosy), American trypanosomiasis, African trypanosomiasis, cysticercosis, dracunculiasis, echinococcosis, foodborne trematode infection, lymphatic filariasis, onchocerciasis, schistosomiasis and soil-borne helminth infection, as well as leishmaniasis. Public health problems are also included, such as the human immunodeficiency virus (HIV)—which causes the acquired immunodeficiency syndrome (AIDS)—tuberculosis, malaria and other neglected infections. Thus, it is a heterogeneous group of diseases with serious social consequences, due to their high capacity for morbidity.

NTDs affect more than one billion people worldwide, with greater severity in individuals submitted to socioeconomic vulnerability, generally belonging to underdeveloped or emerging countries. As a result, social, public health, productivity and life quality problems are intensified, particularly among marginalized populations. Amid the secondary factors related to the persistence of NTDs, the following stand out: (a) gaps in science and research, for presenting insufficient knowledge, non-related to these diseases, (b) market factors, such as high-cost drugs, in existing or developing vaccines, and (c) gaps in public health, represented by poor access to medicines and treatments as a consequence of failures in administrative planning, even when such services are offered for free [4].

From this perspective, NTDs represent an enormous challenge for Brazil and other nations, since they are considered the infections with the highest levels of occurrence—such as cutaneous or visceral leishmaniases, whether endemic or not. These diseases are heightened by their multiple forms of clinical manifestations, which depend on the pathogenicity of the infecting species. Its characteristics modalize leishmaniasis among its subclinical infections, with localized or disseminated microlesions, whether asymptomatic or not. In addition, wounds can spontaneously heal or progress from milder to more severe forms, which is why the WHO recommends priority treatment for this disease [1].

Leishmaniasis is considered an important public health problem in the world and is one of the six most important infectious and parasitic diseases of NTDs, with an annual incidence of 2 million cases of different clinical forms, representing a risk to 350 million people in developing countries [1,2].

Leishmaniases are diseases caused by the protozoan of the genus Leishmania that are marked by great clinical pleomorphism. The parasites that cause them are included in the subkingdom Protozoan, phylum *Sarcomastigophara*, order *Kinetoplastida* and family *Trypanosomatidae*, which are responsible for morbidity and mortality in several countries in tropical zones. Seven species that cause tegumentary leishmaniasis have already been identified in Brazil, six from the subgenus *Viannia* and one from the subgenus Leishmania. The three main species are: *L. (V.) braziliensis, L. (V.) guyanensis* and *L. (L.) amazonensis,* and more recently, the species *L. (V.) lainsoni, L. (V.) naiffi, L. (V.) lindenberg* and *L. (V.) shawi*, identified in states in the North and Northeast regions [1].

Leishmaniases have factors that are increased by the virulence and dissemination of the protozoan, which depend on the host–parasite relationship. This is because the manifestation of the disease is not only correlated with the species of parasite involved, but also with the susceptibility of the host, assessed by its immune response [5]. Furthermore, the protozoan–susceptibility interaction is responsible for the strength of leishmaniasis, making it resistant to available therapeutic treatments, which are scarce and restricted to two lines: antimonials and non-antimonials. Likewise, the lack of a vaccine and the high toxicity rate of the treatment demonstrate the need for new antileishmanial drugs [6].

Due to the incipience of first-line medicines and considering the potential of the Amazon, research in the areas of phytochemistry and ethnopharmacology are increasingly important to expand knowledge in strategic areas related to Research, Development and Innovation (RD and I) about traditional medicines. Hence, it is possible to create prototypes of promising new chemotherapeutic agents with anti-leishmanial activity, with minimized or non-existent side effects.

Amid the promising Amazonian plants for such objectives, we highlight the species *Trattinnickia rhoifolia (T. rhoifolia)*, an angiosperm tree present in the biogeographic region that comprises Central America that belongs to the *Burceraceae* family, of the *Canarieae* tribe [7,8].

The species *Trattinnickia rhoifolia* is used by traditional peoples to cure sore throats and skin lacerations, as well as to treat and prevent tumors and leukemia. To this end, the most used parts of the plant are the trunk bark, resin and leaves [8].

For the genus *Trattinnickia*, studies have already been carried out on photochemical mapping in the bark of the species *Trattinnickia peruviana*, which indicated the presence of lichen xanthone and triterpenes. Furthermore, triterpenes and sesquiterpene lactones were isolated in the resin of the species *Trattinnickia aspera* [7,8,9]. Additionally, the compounds podocarpus-flavone A and biflavonoid were identified in the leaves of *Trattinnickia glaziovii* [7,8,9].

Regarding the species *T. rhoifolia*, the focus of this study, triterpenes, monoterpenes, amentoflavones, sterols and sesquiterpene lactones have already been identified in different parts of the plant, such as resin, leaves and in the bark of its trunk [10,11].

It is important to present the phytochemical mapping of the species *Trattinnickia rhoifolia* Willd and consequently of the genus *Trattinnickia*, which is a promising source for obtaining biopharmaceuticals from the production of secondary plant metabolites and, as a result, represents an opportunity in the field of drug innovation. For this reason, it is essential to know the research that addresses its main characteristics, such as taxonomic, ethnopharmacological and phytochemical studies, in order to direct future biological tests based on the bioactive action of the species *Trattinnickia rhoifolia* Willd.

In this sense, this study constitutes, methodologically, an analytical bibliographical review related to the mapping of secondary metabolites found in the genus *Trattinnickia* and the studies on their pharmacological potential. Data collection was carried out from September 2020 to May 2021, using the following databases: CAPES journals, PubMed, Science Direct from Elsevier, Wiley Online Library, Springer-Nature, Taylor and Francis, BMC, Hindawi, Scielo, ACS—American Chemical Society, and Google Scholar, as well as the scientific article and patent databases “The LENS” and “ORBIT Intelligence”.

The inclusion criteria for this work were: original articles and exclusive dissertations of the genus and species studied, with full text available in Portuguese, English or other languages. Exclusion criteria were: abstracts, online sites without scientific sources, incomplete texts, unrelated and repeated articles.

As for the search strategy, the descriptive words used in this work were: family *Burseraceae*, genus *Trattinnickia* and species *Trattinnickia rhoifolia* Willd, correlated with secondary metabolites and their pharmacological potential. The articles were selected by reading the titles and abstracts of publications, associated with the Boolean descriptor “AND”, in order to refine the samples.

Therefore, the innovation present in this study consists in elucidating the potential use of the species *Trattinnickia rhoifolia* Willd, mainly due to reports of the use of the resin, bark and leaves of the plant by traditional peoples of the Amazon Region and, in this sense, assisting future studies that seek to understand and perform the chemical mapping of that species, highlighting its high pharmacological potential.

Hence, the review contributes to direct future studies that also prove the importance of evaluating the biological activities of the species *Trattinnickia rhoifolia* and address the scarcity of studies on the classes of metabolites present in the studied species, which comes from the *Burseraceae* family.

## 2. Taxonomy of the *Burseraceae* Family

The *Burseraceae* family has 19 genera and more than 700 species [7,8], composed of trees and shrubs. This group of plants, distributed in tropical and subtropical regions, is characterized by the production of aromatic oleoresins. More recently, APG IV 2016 performed an update on the taxonomic status of these angiosperm plants, recognizing several new orders. Among them, the following stand out: *Boraginales*, *Dilleniales*, *Icacinales*, *Metteniusiales* and *Vahliales*. Besides, this system suggests the inclusion of *Burseraceae* in the order *Sapindales*, class *Dicotiledoneae* and subclass *Rosidae* (APG IV 2016) [7].

Of the 19 genera of *Burseraceae* distributed in South America, only 6 are native (*Bursera, Crepidospermum, Hemicrepidospermum, Paraprotium, Tetragastris* and *Trattinickia*). In their turn, the other two (*Dacriodes* and *Protium*) were introduced and are representative in the Amazon Region [9]. One of the most expressive properties of *Burseraceae* is the natural production of aromatic oleoresins, which are used regionally in alternative medicinal treatments and in the ethnopharmacology of Amazonian traditional peoples. The plant genera *Dacryodes* and *Protium* are popularly known as: “almescla”, “breu-branco”, “breu-preto”, “breu-vermelho”, “breu-terra”, “breu-limão”, “ breu-manga”, “caraña”, “copal”, “copal-ouro”, “copal-negro”, “elemi”, “manila-elemi”, “frankincenso”, “gugul”, “maaliol”, “mirra” and “okume” [10,11,12].

## 3. Chemical Characteristics of *Burseraceae*

The mapping of the chemical compounds of the *Burseraceae* family is concentrated in the oil–gum–resin, from which fractions considered volatile are usually obtained [9,12]. In principle, the volatilization of aromatic compounds in the exudate is responsible for the yellowish appearance and viscous liquid, which occurs because of the natural oxidation process (oxidized eudesmane). During deposition on the surface of the trunks, the resin acquires a grayish color and a greater degree of ductility—that is, it becomes more resistant [12,13].

The manipulation of the resin is historically common among the traditional peoples of the Amazon, who use it in the manufacture of varnishes and paints, along with boat-caulking. The aromatic constituents that are present in the resin, bark and branches of the plant are widely used through combustion, as well as for lighting the homes of traditional Amazonian peoples, as repellents for undesirable insects and also in religious rituals [9,10,11,12,13]. Concerning food, the family has species that produce almonds—some with edible mesocarp, which represent sources of light fatty oil and have a pleasant taste. Its fruits contain 10% more sugar and produce 25% of this oil, indicated as a substitute for olive oil [13,14].

The chemical characterization studies of the *Burseraceae* family focus on the constituents present in its leaves, of which the monoterpenes represent the dominant class of compounds, distributed in limonene, α-phellandrene, *p*-cimene and their monocyclic derivatives. Aldehydes, ketones and acids are also present, though less frequently [14,15]. In pharmaceutical correlation, studies relevant to this class of compounds are involved in anti-acaricide, anti-inflammatory, anti-leishmania and antiviral activities [12,13]. There are studies in the literature with bicyclic monoterpenes such as thujan, pinane and canphane, which are associated with antifungal activity against *Cryptococcus neoformans* var. *Neoformans*—a systemic human fungus of high prevalence in immunosuppressive patients [13,16].

There are similar chemical profiles that identify over 30 sesquiterpenes in the *Burseraceae* family. The most common and complex are in the genera *Canarium* and *Commiphora*, except in the species *Boswellia carterii*. In those cases, cadinene is isolated in essential oils obtained from the leaves, as the substance is found in lower concentrations in other parts of some species of this family [15,16]. From the genus *Canarium*, the elemol group was also isolated, which constitutes the synthesis of germacra (10), 4 diene, widely used to combat Phlebotomus—the genus to which the agents that transmit malaria and leishmaniasis belong [13,17,18]. This justifies the traditional use of the resin as a repellent.

On the other hand, essential oils from *Protium* leaves are rich in cariophilia, which is generally ubiquitous, and in sesquiterpenes, described as bactericides for the etiological agents that cause acquired respiratory infections, such as otitis, sinusitis and pneumonia [17,18,19]. Some sesquiterpenes such as cubebenol and β-Cubebene, involved in the mechanisms of inflammatory and immune responses, have also been isolated from *Burseraceae* [18,20]. In studies related to the tribe *Canarieae*, isolations of furanosesquiterpenes are described, which are furanogermacrane skeletons, forming 2-acetyl-8-12-epoxygermacra-1, (10), 4,7,11 tetraene and 2-methyl-8-12-epoxygermacra-1, (10) 4,7,11-tetraene. It is noteworthy that the active ingredients of these molecules are sources used in the composition of antimalarials [13,18,19], which is why they are considered the most promising class of compounds for immunomodulatory activities. Regarding antitumor substances, we can mention the example of the diterpenoid taxol, which has already been applied in the treatment of breast and uterine cancer, with less invasive results in immunocompromised patients [14,18,20].

In *Burseraceae* resins, the existence of tetracyclic and pentacyclic triterpenes is identified. These compounds are responsible for important characteristics, such as oxidation, due to the presence of tetracyclic tirucallane in the C-17 side chain, which is found as elemolic acid. In the scientific literature, the anti-inflammatory activity and the inhibition of the production of nitric oxide are also reported, in addition to in vitro antitumor activity [14,19,20]. This class of compounds was isolated from *Canarium schweinfurthii* and *Dacriodes eludis*, species of the tribe *Canarieae*, in which the study related to the respective compounds proved the existence of anti-inflammatory, antimicrobial, antiplasmodic, antiulcerogenic, anticariogenic, antiviral, anti-HIV, hepatoprotective, cardioprotective and analgesic activities [14,21,22,23].

In their turn, flavonoids are essential in plant systematics, but there were few technical-scientific reports found about these compounds in *Burseraceae*. Kaempferol, quercetin and their respective derivatives were identified in *Protium* and *Commiphora* [15,16,17,18,19,20,21,22,23,24]. There are, consequently, few reports related to the identification of coumarin in *Burseraceae*. However, coumarinolignoids and propacin have already been identified and isolated from *Protium opacum*—compounds that have hepatoprotective, immunomodulatory and anticancer activities [14,25,26]. Among medical reports, the healing and anti-inflammatory activities of the leaves of the species *Protium helio* and *P. icicariba* stand out [27].

Chemical research on the stem of the botanical family *Burseraceae* focuses mainly on pentacyclic triterpenes. The most abundant are the α-amyrin and β-amyrin isomers [28], which have anti-inflammatory, antinociceptive, gastroprotective, hepatoprotective and antipruritic pharmacological activities, among others, [11,13,20], with recognized low toxicity [20,29].

The studies carried out using stems, branches and bark of the species *P. robustum*, *P. trifoliatum, P. nitidifolium* and *P. subserratum*, collected in the Amazon region, identified sesquiterpenes in a higher percentage, as well as the following compounds: spatulenol, caryophyllene oxide, Khusimone, α-copaen-4-ol, and in smaller amounts, α-cubeben, α-copaene, trans-caryophyllene, α-trans-bergamothene, γ-muurolene, α-muurolene, γ-cadinene [14,20], α-calakorene, spathulenol, caryophyllene oxide, β-copaen-4-α-ol, khusimone, 1-epi-cubenol, γ-eudesmol and α-muurolol, with eighteen sesquiterpenes having the highest percentage of hydrocarbon sesquiterpenes (35.97%): α-cubeben, αcopaene, β-cubeben, *trans*-caryophyllene, α-elemene, α-gurjunene, germacrene D, α- and β-selinene, *cis*-calamenene, *trans*-calamenene and cadalene [14,20,28,29]. The other sesquiterpenes are oxygenated, totaling 24.20% of the essential oil composition [20,28,29].

The literature also describes the presence of aromatic compounds such as coumarins, phenylpropanoids, flavonoids and, to a greater extent, lignans, which demonstrates the biological potential of the family [11,18]. There are few reports related to the identification of coumarins in *Burseraceae*, but coumarinolignoid and propacin have already been identified and isolated from the species *Protium opacum* [18].

### Characteristics of the Species Trattinnickia Rhoifolia Willd

**Figure 1:**
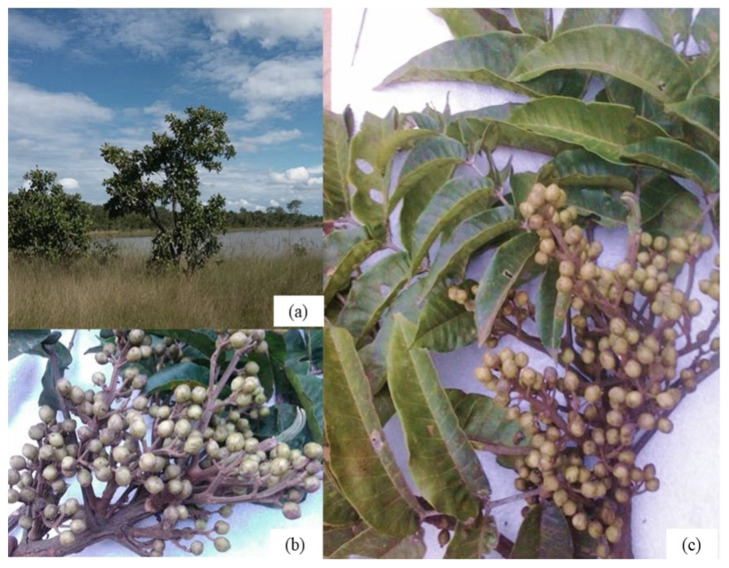
Photo: (**a**) shrub of *Trattinnickia rhoifolia,* (**b**) seeds and (**c**) alternate compound leaves.

The species *Trattinnickia rhoifolia* Willd is popularly known in the Amazon region as “Breu sucuruba”. Its native origin is not endemic to Brazil, but *T. rhoifolia* Willd has an Amazon phytogeographic occurrence [8,9,30]. The studied group belongs to the kingdom *Plantae*, phylum *Tracheophyta*, class *Magnoliopsida*, order *Sapindales*, family *Burseraceae*, genus *Trattinnickia* and species *Trattinnickia rhoifolia*, which has wide ethnopharmacological use by indigenous peoples (Figure 1) [8,9,18,31].

The species *Trattinnickia rhoifolia* Willd is characterized by 32 m tall trees with 1.00 m DBH (diameter at 1.30 m from the ground), digitized base and cylindrical shaft. In addition, it has a grayish rhytidome with depressions and detachment in small woody plaques, which leave light streaks, lenticels are present, 2 mm thick dead brown skin and 1 cm thick bright orange skin. Orange sapwood has transparent exudation with a “breu” smell [10,12,18].

The distribution of the aromatic resin present in the species *Trattinnickia rhoifolia* Willd is more predominant in the upper parts of the species, so that only the aromas attributed to the presence of terpenes are uniform. Due to this characteristic, the resin is used to combat some species of insects from the *Psicodidae* and *Culicidae* families. As a result of this practice, there is a reduction in the prevalence of malaria and leishmaniasis in indigenous villages and riverside communities [8,9,30,32,33]. For this reason, species of the genus *Trattinnickia* are widely known in the Amazon region by popular medicine as repellent, analgesic, anti-inflammatory, expectorant and healing plants [11,13,20,34].

Nonetheless, scientific research that demonstrates its performance is incipient or almost non-existent, as illustrated by the scarcity of studies on the action mechanisms of species of the genus *Trattinnickia* in parasitic diseases such as leishmaniasis [33].

## 4. Potential Use of the Species *Trattinnickia rhoifolia*

The *Burseraceae* family has extensive chemical mapping and demonstration of its pharmacological properties, proven by its anti-inflammatory, antimicrobial, antiparasitic and antifungal activities. However, there are few studies regarding the identification of compounds for the genus *Trattinnickia* (Table 1) [17,34,35]. Likewise, focused research and phytochemical mapping of the species *Trattinnickia rhoifolia* Willd are scarce, although it is widely used in Amazonian folk medicine for the treatment of several diseases, including leishmaniasis, due to its synergistic, analgesic, anti-inflammatory and curative effects [35,36].

In the chemical composition of oil-resins of the species *Trattinnickia rhoifolia*, there is a predominance of sesquiterpenes, such as α- and β-selinene, β-caryophyllene, β-bisabolene and α-humulene. The latter has anti-inflammatory and analgesic properties, while the penultimate is described in the literature as anti-edemic, anti-inflammatory, bactericidal and insect-repellent [18,20,35,36,39].

Furthermore, other biological activities are reported on the species, related to treatments for leukemia and carcinomas [36,37], in addition to hepatoprotective activity [38]. Compounds isolated from *T. rhoifolia* Willd in Colombia showed mainly β-bisabolene [38,39] as a substance with strong fungicidal and antimicrobial powers, being efficacious against the bacteria *Staphylococcus aureus* [39], which is associated with several infections: from boils to septicemia (sepsis), including endocarditis (cardiac infections) and abscess [40,41,42]. Along with β-bisabolene, amentoflavone is also identified in the leaves of *T. rhoifolia* Willd and it is related to delay in cell degradation and aging, to anticancer activity and to other biofunctions.

In another study, *T. rhoifolia* presented β-sitosterol in in vitro tests, in which it was observed that pretreatment with the use of human breast cancer cells was able to inhibit cell invasion. Additionally, an in vivo test showed a reduction in the number and size of tumors in lymph node and lung metastases [43]. In the pharmacological activities of β-sitosterol, it is suggested to consume a dose of milk of 300 mg to 5 g for lowering high cholesterol, so higher doses are indicated for symptoms related to prostatic hyperplasia [18].

**Figure 2:**
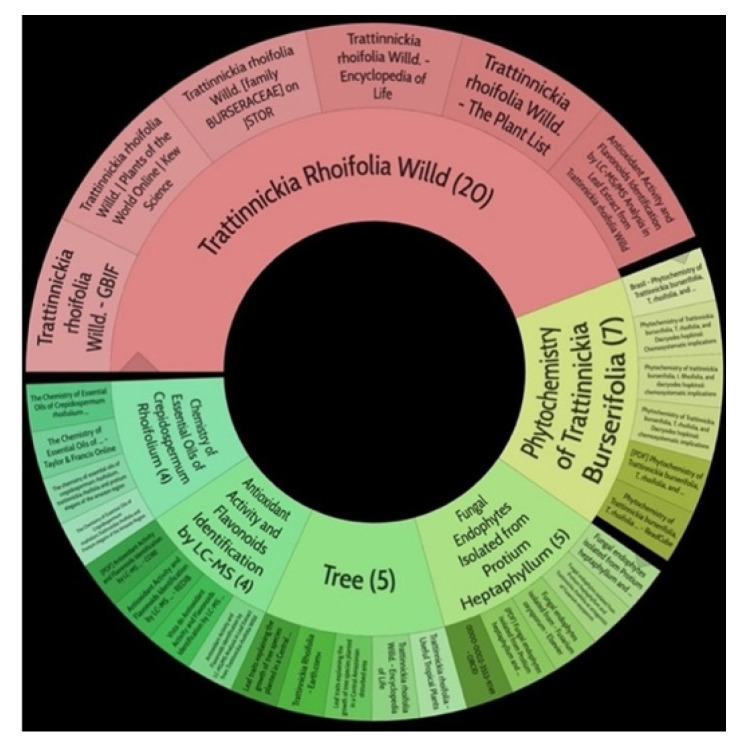
Pharmacological and phytochemical studies of the species *Trattinnickia rhoifolia* clustered by the Carrot^2^ search engine in 2021.

Moreover, diterpene was found, a compound for which the activities are described in the literature as antiparasitic and antimicrobial (Figure 2).

Studies involving eicosane and nonacosane produced important antiparasitic effects against strains of *Leishmania maior, L. donovani* and *Trypanosoma brucei*, in addition to cytotoxic activity against several types of tumor cells [33,39]. In this context, it is important to report that research involving hydroalcoholic extracts from the leaves of *Trattinnickia rhoifolia* Willd showed stimulating activity in the treatment of cancer, with prevention of tumors and leukemia. Tests using leaf extracts produced with dichloromethane and acetone showed a moderate effect on the P388 leukemia cell in mice (experimental lymphocytic leukemia, originally induced in DBA/2 mice with methylcholanthrene) [39,44].

In studies on extracts from the leaves of *Trattinnickia rhoifolia*, the existence of amentoflavan was identified, also found in oil-resins, 5α, 6α epoxy-β-sitosterol (IV) and 5β, 6β-epoxy-β-sitosterol (V) [14,46]. Besides, the researchers observed evidence of inhibition of the adenosine receptors GABA-A and B, 5-hydroxytryptamide, central benzodiazepine, forskolin, inositol triphosphate and MAO A and B enzymes, which results in an antidepressant response [39].

From this perspective, the authors have reported that amentoflavone acts in favor of the inhibitory activity of cAMP phosphodiesterase, among other enzymes. The process of inhibiting these mechanisms results in tissue regeneration [39,45]. Furthermore, it is possible to find experiments with amentoflavone linked to antioxidant, antifungal and anti-inflammatory activities. Therefore, this molecule also represents a promising compound in anti-HIV activities, which establishes this species among the most promising in the phytochemical profile for new drugs [39,46].

## 5. Conclusions

The extensive bibliographical research revealed that the genus *Trattinnickia*, which belongs to the *Burseraceae* family, represents an important ethnopharmacological source widely used by traditional peoples in the Brazilian Amazon due to its cultural and cosmological attributes in the treatment of Neglected Tropical Diseases, related to anti-inflammatory processes and antiparasitic and repellent functions.

The reviewed scientific literature indicates that the biological activities of *Trattinnickia rhoifolia* are similar to the traditional use of the species, which associates the following properties to the “*breu scuruba*”: anticancer, anti-inflammatory, antimicrobial, neurological and antiparasitic, all attributed to the phytochemistry of the genus.

The main classes of secondary metabolites, already characterized both in number of compounds and in pharmacological and environmental importance, are: alkaloids, terpenoids, sesquiterpenes, flavonoids, biflavonoids, oxyphytosterols and volatile oils. However, scientific support is restricted to mappings available for Asian and African genera, and studies on the subject are limited to these continents, even though the family is distributed in several tropical and subtropical regions of the world.

Although the results of this review are very promising for the use of the genus *Trattinnickia* as a multipurpose drug related to its pharmacological groups, there are several limitations in the current literature, especially with regard to studies for the species *Trattinnickia rhoifolia* Willd. On the other hand, popular knowledge bases are important to recognize that traditionally used extracts of the species *Trattinnickia rhoifolia* Willd can be effective not only when isolated or in direct use, but can also represent new and promising alternatives, with modulating effects when used in combination with other herbs or drugs.

Therefore, studying the species *Trattinnickia rhoifolia* Willd is essential for further work because of the importance of the results raised here, especially given the pharmacological potential of the group, which should be better explored—especially concerning the use of the species for its anti-inflammatory and analgesic activities, as reported by traditional communities in the Amazon region and presented in this article.

## Figures and Tables

**Table 1:** Secondary metabolites found among the species *Trattinnickia rhoifolia* and their respective pharmacological activities.

Parts Used	Isolated or Characterized Constituents		Pharmacological Activities	
Aerial	Sesquiterpenes: α-selinene (**1**), β-selinene (**2**), β-bisabolene (**3**) Monocyclic sesquiterpene: α-humulene (**4**) Bicyclic sesquiterpene: β-caryophyllene (**5**)		Anti-inflammatory, analgesic, anti-edemic, bactericide and insecticide [20,35,37,38,39]	
Aerial oil-resin	Sesquiterpenes: β-bisabolene (**3**)		Fungicidal and antimicrobial tests show efficacy against *Staphylococcus aureus* [40,41]	
Aerial leaves	Linear alkane: Eicosane (**6**); nonacosane (**7**) Alkane: octadecane (**8**), octacosane (**9**), nonacosane (**7**), tetrapentacontane (**10**)		Antiparasitic against strays of *Leishmania maior*, *L*. *donovani*, *Trypanossoma brucei*, cytotoxic activity against various types of tumor cells [33], healing [38,42], anti-inflammatory and analgesic, anti-edemic, anti-inflammatory, bactericide and insecticide, fungicide [38,39] and antimicrobial [39,43]	
Leaf	Biflavonoid: amentoflavane (**11**), Oxyphytosterols: 5α, 6α epoxy-β-sitosterol (**12**), 5β, 6 β-epoxy-beta-sitosterol (**13**)		Indicative of inhibition of the adenosine receptors GABA-A and B, 5-hydroxytryptamide, central benzodiazepine, forskolin and inositol triphosphate, as well as MAO A and B enzymes [42]. Inhibition of these enzymes results in an antidepressant response [39]	
Leaf	Biflavonoid: amentoflavone (**11**)		Inhibitory activity of cAMP phosphodiesterase, [39,41,44] antioxidant, antifungal and anti-inflammatory activities [45,46], as well as indicative anti-HIV activities [39,47]	
Resin	Monoterpenes: α-pinene (**14**), camphene (**15**), sabinene (**16**), β-pinene (**17**), α- phellandrene (**18**), α-terpinene (**19**), *p*-cymene (**18**) (**20**), β-phellandrene (**21**), γ-terpinene (**22**), α-terpinolene (**23**), *trans*-α-di- dihydroterpineol (**24**), terpinen-4-ol (**25**), *p*-cymen- 8-ol (**26**), α-terpineol (**27**)		Anti-inflammatory, inhibition of nitric oxide production, in vitro antitumor [48,49]	
Leaf	Fatty acids: pentadecanoic acid (**28**); nonadecanoic acid (**29**); methyl-11,14,17-eicosatrienoate ester (**30**)		Antioxidant, antifungal and anti-inflammatory [48]	
Leaf	Sesquiterpenoid: biatractylolide (**31**)		Anti-tumor and antioxidant, reduction of AChE activity, improvement of brain and memory capacities [47]	
Resin	Triterpenes: ursane (**32**), α-amrinone (**33**),α-amyrin (**34**), 3-epi-α-amyrin (**35**), 3α,16β-dihydroxyurs-12-ene (**36**), β-amrinone oleananes (**37**), β-amyrin (**38**), 3-epi-β-amyrin (**39**), 3α,16β-dihydroxyolean-12-ene (**40**), 3α-hydroxytyrucal-8,24-dien-21-oic tirucallan acid (**41**), 3α-hydroxytirucal-7,24-dien-21-oic (**42**) 3-oxotyrucal-8,24-dien-21-oic (**43**), dammarane dammarenediol-II (**44**), 3α,20(S)-dihydroxydamar-24-ene (**45**), 3β-phenylacetoxyurs-12-ene (**46**), 3β-phenylacetoxyolean-12-ene (**47**) and 3β,16β,11α-trihydroxyurs-12-ene (**48**) Monoterpene: 2(S*)-phenylacetoxy-4(R*)-p-mentha-1(7),5-diene (**49**)		anticancer, anti-inflammatory, antileprotic, antiviral, antibacterial, antifungal, antidiuretic, giardicide and inhibition of acetylcholinesterase enzymes [48,49]	
**α-selinene** 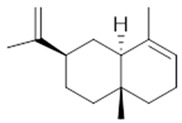 (**1**)	**β-selinene** 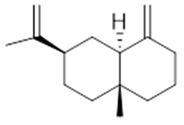 (**2**)	**β-bisabolene** 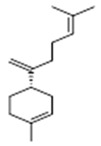 (**3**)	**α-humulene** 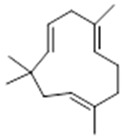 (**4**)	**β-caryophyllene** 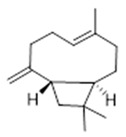 (**5**)
**Eicosane** 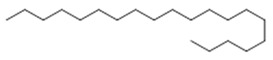 (**6**)	**Nonacosane** 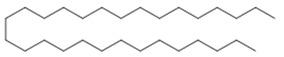 (**7**)		**Octadecane** 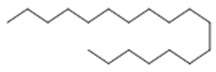 (**8**)	
**Octacosane** 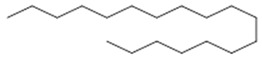 (**9**)	**tetrapentacontane** 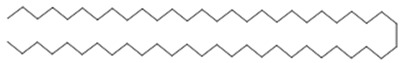 (**10**)			
**amentoflavone** 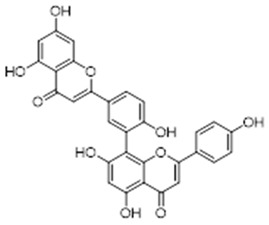 (**11**)	**5a, 5α, 6α epoxy-β-sitosterol** 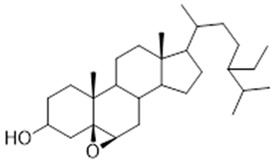 (**12**)		**5β, 6 β-epoxy-beta-sitosterol** 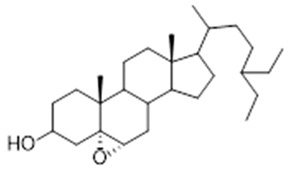 (**13**)	
**α-pinene** 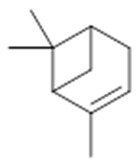 (**14**)	**camphene** 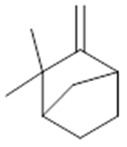 (**15**)	**Sabinene** 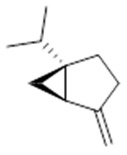 (**16**)	**β-pinene** 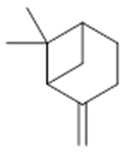 (**17**)	**a-phellandrene** 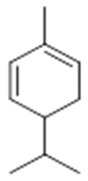 (**18**)
**α-terpinene** 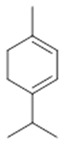 (**19**)	***p*-cymene** 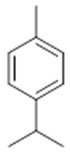 (**20**)	**β-phellandrene** 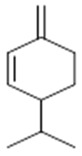 (**21**)	**γ-terpinene** 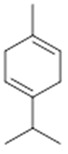 (**22**)	**α-terpinolene** 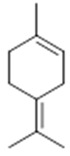 (**23**)
***trans*-α-di-dihydroterpineol** 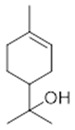 (**24**)	**terpinen-4-ol** 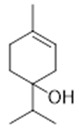 (**25**)		***p*-cymen-8-ol** 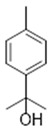 (**26**)	**α-terpineol** 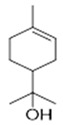 (**27**)
**pentadecanoic acid** 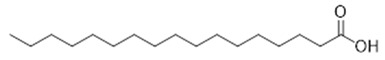 (**28**)			**Nonadecanoic acid** 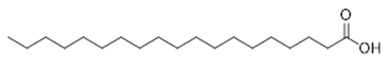 (**29**)	
**methyl-11,14,17-eicosatrienoate ester** 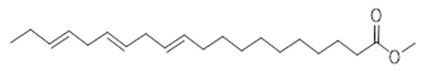 (**30**)			**biatractylolide** 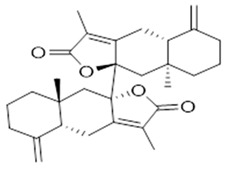 (**31**)	
**ursane** 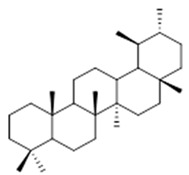 (**32**)	**α-amrinone** 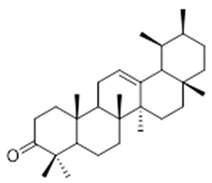 (**33**)		**α-amyrin** 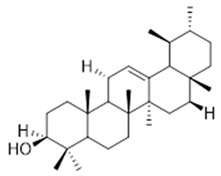 (**34**)	
**3-epi-α-amyrin** 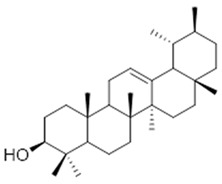 (**35**)	**3α,16β-dihydroxyurs-12-ene** 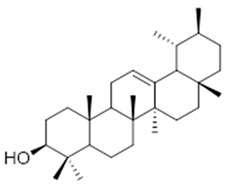 (**36**)		**β-amrinone** 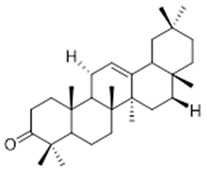 (**37**)	
**β-amyrin** 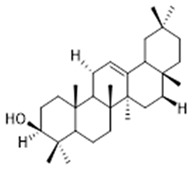 (**38**)	**3-epi-β-amyrin** 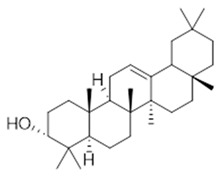 (**39**)		**3α-hydroxytyrucal-8,24-dien-21-oic** 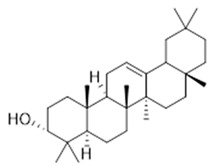 (**40**)	
**3α-hydroxytyrucal-7,24-dien-21-oic** 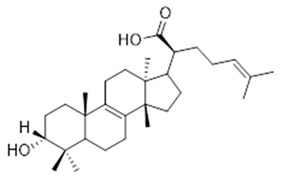 (**41**)	**3-oxotyrucal-7,24-dien-21-oic** 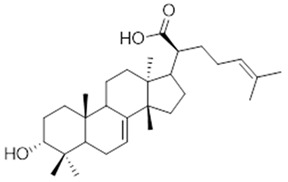 (**42**)		**3-oxotyrucal-8,24-dien-21-oic** 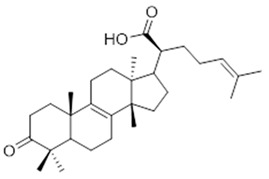 (**43**)	
**dammarane dammarenediol-II** 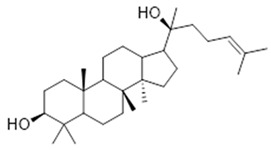 (**44**)	**3α,20(S)-dihydroxydamar-24-ene** 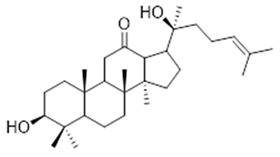 (**45**)		**3β-phenylacetoxyurs-12-ene** 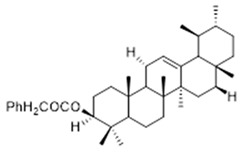 (**46**)	
**3β-phenylacetoxyolean-12-ene** 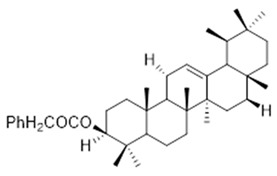 (**47**)	**3β,16β,11α-trihydroxyurs-12-ene** 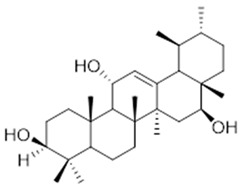 (**48**)		2(S*)-phenylacetoxy-4(R*)-p-mentha-1(7),5-diene 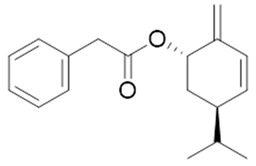 (**49**)	

## Data Availability

Not applicable.
